# How to measure image quality in tissue-based diagnosis (diagnostic surgical pathology)

**DOI:** 10.1186/1746-1596-3-S1-S11

**Published:** 2008-07-15

**Authors:** Klaus Kayser, Jürgen Görtler, Konradin Metze, Torsten Goldmann, Ekkehard Vollmer, Masoud Mireskandari, Zdravko Kosjerina, Gian Kayser

**Affiliations:** 1 UICC-TPCC, Institute of Pathology, Charite, Berlin, Germany; 2 IBM DeepComputing, Brussels, Belgium; 3 Institute of Pathology, University of Campinas, Campinas, Brasil; 4 Institute of Pathology, Research Center Borstel, Borstel, Germany; 5 Department of Pathology, Iran University of Medical Sciences, Tehran, Iran; 6 Institute of Pathology, University Novi Sad, Novi Sad, Serbia; 7 Institute of Pathology, University Freiburg, Freiburg, Germany

## Abstract

**Background:**

Automated image analysis, measurements of virtual slides, and open access electronic measurement user systems require standardized image quality assessment in tissue-based diagnosis.

**Aims:**

To describe the theoretical background and the practical experiences in automated image quality estimation of colour images acquired from histological slides.

**Theory, material and measurements:**

Digital images acquired from histological slides should present with textures and objects that permit automated image information analysis. The quality of digitized images can be estimated by spatial independent and local filter operations that investigate in homogenous brightness, low peak to noise ratio (full range of available grey values), maximum gradients, equalized grey value distribution, and existence of grey value thresholds. Transformation of the red-green-blue (RGB) space into the hue-saturation-intensity (HSI) space permits the detection of colour and intensity maxima/minima. The feature distance of the original image to its standardized counterpart is an appropriate measure to quantify the actual image quality. These measures have been applied to a series of H&E stained, fluorescent (DAPI, Texas Red, FITC), and immunohistochemically stained (PAP, DAB) slides. More than 5,000 slides have been measured and partly analyzed in a time series.

**Results:**

Analysis of H&E stained slides revealed low shading corrections (10%) and moderate grey value standardization (10 – 20%) in the majority of cases. Immunohistochemically stained slides displayed greater shading and grey value correction. Fluorescent stained slides are often revealed to high brightness. Images requiring only low standardization corrections possess at least 5 different statistically significant thresholds, which are useful for object segmentation. Fluorescent images of good quality only posses one singular intensity maximum in contrast to good images obtained from H&E stained slides that present with 2 – 3 intensity maxima.

**Conclusion:**

Evaluation of image quality and creation of formally standardized images should be performed prior to automatic analysis of digital images acquired from histological slides. Spatial dependent and local filter operations as well as analysis of the RGB and HSI spaces are appropriate methods to reproduce evaluated formal image quality.

## Introduction

The technological progress in image acquisition, transfer, and display prompted surgical pathologists to investigate and implement new image viewing techniques in their daily work. Telepathology, which is the transfer and viewing of macroscopic and microscopic images at a distance, was fully established at the beginning of the 1990s followed by the construction of specific telemedicine systems such as the iPATH or UICC-TPCC at the beginning of this century [1-3]. Only a few years later the technology of digitizing complete glass slides is commercially available as well as internet-based automated image measurement systems such as EAMUS™ [4]. The introduction of video assistance in tissue – based diagnosis is only a question of time, and some institutions of pathology report that they are already performing daily routine diagnosis with solely electronically viewed slides in a test phase [5-10].

This scenario requires a formal and reproducible analysis of the image quality to be viewed electronically due to practical and also legal reasons. The definition of image quality is, however, not simple. The viewing of images induces, in addition to their optical properties, emotions that are not completely associated with the physical components: Quite independent from clearly defined terms, such as contrast, resolution and focus, pathologists often state that some images are easy to view at and others are not [8]. Thus, to extract visual information from an image depends upon additional factors that are probably related to the "biology" content in addition to the optical features. The analysis of the biological properties and the access to their recognition depends on terms of texture and – in addition – object representation in the corresponding image. This has been discussed in detail in [11] and [12]. For example, the measurement of a texture requires a certain amount of image pixels. Usually, images below 256 × 256 pixel area are judged to be less "convenient" as well as objects covering less than about 50 – 100 pixels in area [11].

On the other hand, formal parameters of image quality contribute to recognition of image information too. Especially the application of automated texture and object analysis requires formally standardized images prior to the image examination [2-4,11].

In this article we describe the theoretical background and our experiences in applying formal standardization in microscopic images prior to automated information analysis. Digitized images obtained from H&E stains, performed immunohistochemistry, and applied fluorescent dyes served for this investigation.

## Materials and methods

The preparation of histological glass slides is subject to quality influences that are induced by tissue fixation, embedding, and cutting procedures, followed by influences of stains and coverglass fixation, to name some of them. Adjustment of the microscope, illumination or brightness of light transmission, selection of fields of view at different magnifications, quality of mounted camera and its position play an additional role that influence the quality of the acquired image. How to measure and correct these parameters?

Computerized tissue-based diagnosis uses at least two different image information sources which are called a) texture and b) object. Analysis of spatial distribution of grey values per pixel reveals texture information, and that of grey values per biological object, object information [2-4,11]. Gray values in one combined or several colour spaces serve as main information source. The less they depend on information independent image parameters such as predefined size, maximum grey value level, or external artefacts, the more efficient is the information analysis. We can distinguish the basic image size in absolute and in relative numbers, absolute in number of image pixels, relative in number of pixels that form an object or that are contributing to a texture. Objects can only be detected if they can be distinguished from non-object areas. This requires the detection of their boundary, for example of the adequate grey value maxima. In practice the boundaries can be visualized by image differentiation (gradient images).

In aggregate, the minimum of image transformations to obtain a standardized image includes a) shading, b) grey value interval, and c) grey value level correction. Correction of shading results in equal distributed illumination and brightness, that of grey value interval in equal distributed grey value histogram, and that of grey value level in expanding grey values adjusted to the maximum possible one. These standardized images might not be of sufficient quality permitting efficient object detection. Thus, the same standardization procedures should be applied to the derived differentiated images (gradient procedures). The obtained distance of the original from the standardized image is obviously a reproducible and objective measure of image quality of grey value image or colour channel.

Microscopic images usually consist of three colour spaces, most frequently displayed in the red-green-blue (RGB) space [4]. Analysis of colour related light intensity, colour space transformation into the hue-saturation-intensity space, or related spaces is often performed. The relationship between the different colour spaces in intensity distribution reflects the correct colour "temperature" and staining colour. It serves as additional quality parameter too.

Microscopic images should contain objects to be measured. Objects are related to grey value threshold in general, thus the number and areas covered by automated threshold procedures can serve as monitor for image quality related to biological information. We applied the algorithm described by Otsu for these purposes [13].

A series of digital images acquired from H&E stained slides in the Institutes of Pathology, Research Center Borstel, Charite, Berlin, University of Campinas, Campinas, Brasil, University of Freiburg, Freiburg, University of Kerman, Kerman, and University of Novi Sad, Novi Sad, Serbia were subject to the described algorithm. In addition, images acquired from fluorescent and immunohistochemically stained slides (DAB, PAP) which have been submitted to the Electronic Automated Measurement User System (EAMUS™) have been included in this study. All in all, more than 5000 images have been analyzed and included in the statistical analysis.

## Results

A survey of the applied measurements is depicted in table 1. The distances of shading correction, and grey value normalization differ only minimally between images obtained from different applied stains.

**Table 1:** 

**Stain**	**Number of images**	**Distance shading**	**Distance grey value distribution**	**Distance grey value height**
H & E	1400	49	54	46
DAB	1200	32	39	35
AP	400	38	42	39
DAPI & TEXAS RED & FITC	120	98	75	129

H & E: hematoxilin – eosin; DAB: diaminobencidine; AP: Alcalic phosphatase; DAPI & Texas Red & FITC: tri-colour fluorescent stain

**Figure 1:**
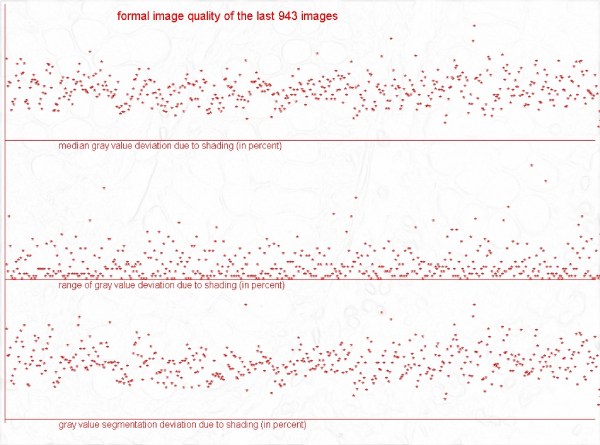
**Gray value levels of shading correction of a total of 943 images, in percent**. a) median grey value (upper line), b) grey value range (medium line), c) grey value segmentation deviation (lower line).

**Figure 2:**
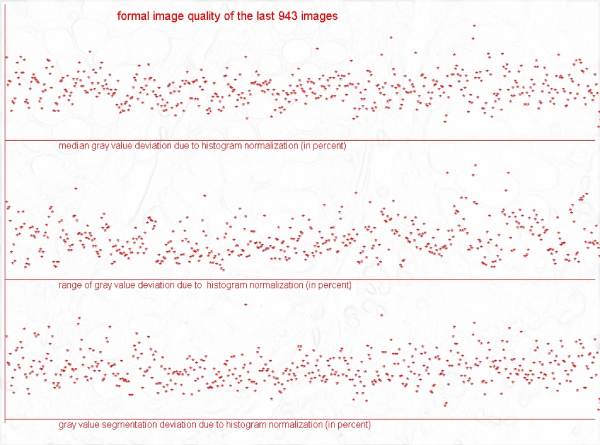
**Gray value levels of shading correction of a total of 943 images, in percent**. a) median grey value (upper line), b) grey value range (medium line), c) grey value segmentation deviation (lower line).

**Figure 3:**
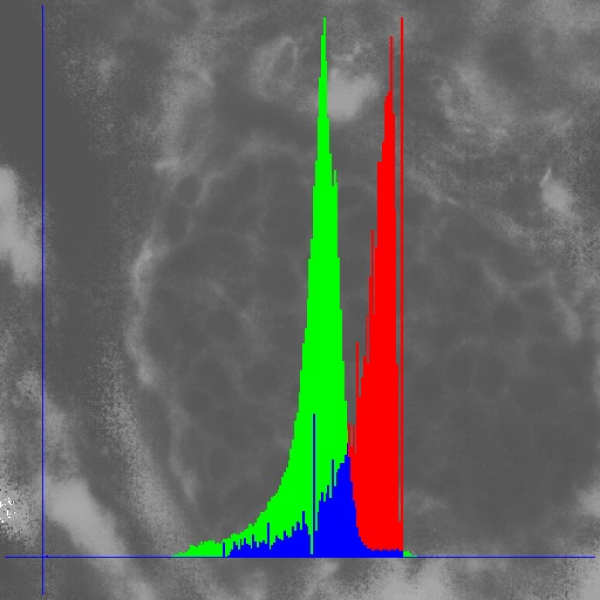
**Gray value histogram standardization of a fluorescent image (DAPI &Texas Red & FITC)**. a) Red colour: Original grey value histogram, b) green colour: standardized histogram.

The results are partly accumulated in serial order, as demonstrated in figure 1 and figure 2. All these images have been acquired and submitted by one institute of pathology only. Extremes in image quality can clearly be seen in both images. A striking example of shading correction of hue-intensity grey value in a fluorescent stained image is shown in figure 3. Thus, even images obtained in the same institution are subject to quite large differences in formal image quality. In images acquired from H&E stained slides, the average number of statistically significant thresholds amounts to 4 – 7 covering an image area of about 60% at the beginning (table 2). Images with a low number of potential segmentation thresholds display a high number of potential, however are not always correct to identify objects (not shown). The common relationship between the different colour spaces in images obtained from H&E stain was similar in those obtained from fluorescent and immunohistochemically stained slides (DAB, PAP). A statistically significant relationship (0.05%) between the colour spaces (hue and saturation) with the intensity space could be obtained in nearly all cases, indicating a reliable staining.

**Table 2:** Explanations: H & E: hematoxilin – eosin; DAB: diaminobencidine; AP: Alcalic phosphatase; DAPI & Texas Red & FITC: tri-colour fluorescent stain

**Stain**	**Number of thresholds**	**Area covered first threshold**	**Area covered 2^nd ^threshold**
**H & E**	3	54%	10%
**DAB**	3	63%	12%
**AP**	3	45%	11%
**(DAPI & TEXAS RED & FITC**	2	35%	5%

## Conclusion

A reliable and standardized quality of digitized images obtained from histological slides is a necessity to further extract information and correlate this information with external data such as diagnosis, prognosis, etc. [12]. For example, in transmission electron microscopy applicable software should provide easy scan control, image digitization, automatic prescale adjustment for acquisition, grey scale histogram generation, grey scale manipulation, image filtering, smoothing, and random colour assignment to grey levels [14]. Especially recent image analysis approaches that include so-called Online Support Vector Classifier (OSVC) require highly standardized images to work on [15]. Similar requirements were noted in quantification aims of synovial tissue, microvascular structures, prostate tissue, or three dimensional tissue analysis and reconstruction [16-20]. Kayser et al. reported about the successful implementation of image quality assessment in the open access immunohistochemistry measurement system EAMUS™ [4]. In addition to the practical experiences theoretical considerations indicate in the same direction, i.e., that image quality assessment and working with standardized images seems to be a prerequisite to further applying artificial intelligence in image analysis [1-4,12,21].

Acknowledged limits of image quality parameters to classifying images of good or less good quality do not exist, to our knowledge. Therefore, a serial analysis of "distances" between original and standardized images seems an adequate procedure in estimating image quality. The results demonstrated in figure 1 and figure 2 indicate that an easy detection of images with great distances between original and standardized image, i.e., images of probably poor quality, is possible. The distances measured on the "original" and "gradient" images do correlate in general; however, good colour and brightness balance do not necessarily indicate good "focus" or object detection properties.

**Figure 4:**
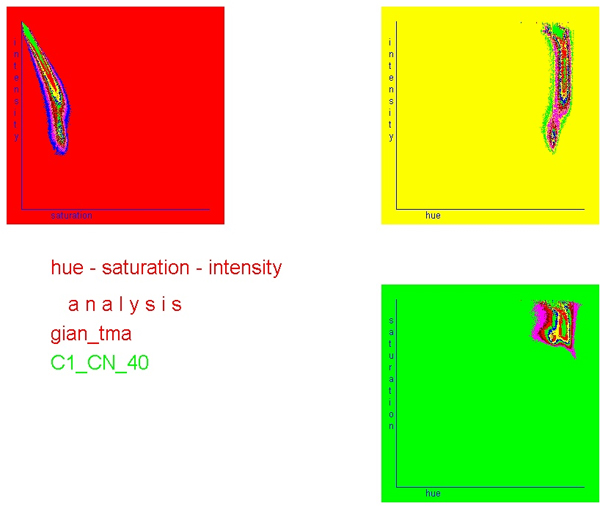
**Correlation of hue – saturation – intensity grey value distribution of an image obtained from H & E stained "slide.** Note the narrow channel of hue – intensity correlation.

**Figure 5:**
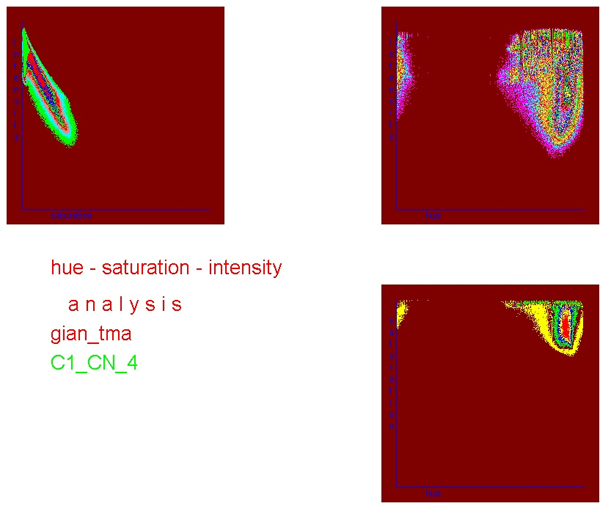
**Correlation of hue – saturation – intensity grey value distribution of an image obtained from H & E stained slide**. Note the quite broad channel of hue – intensity correlation, and two separate clusters (indicators of "poor" image quality).

**Figure 6:**
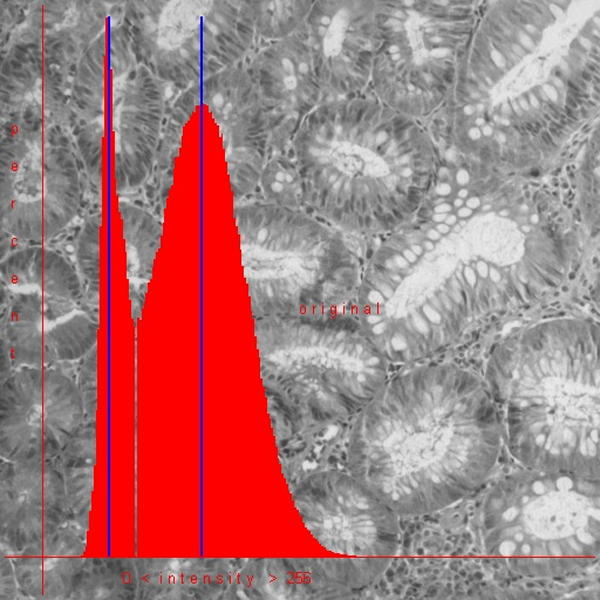
**Intensity histogram of an image obtained from an H & E stained slide after standardization.** Two intensity peaks and one minimum are indicators of appropriate standardization.

The correlation of hue and saturation spaces with the intensity space is closer than that between the different RGB spaces. Images of good quality display only one and strictly closed space in the corresponding figures (see figure 4 and figure 5). In addition, the intensity histogram of good HSI images displays usually only one peak and one minimum (figure 6).

The image measurements reported herein are performed on images of (.bmp) format without any compression mode. The influence of compression on image and tissue based diagnosis quality had been already extensively analyzed by several authors [6,22-25]. A lossy image compression of a ratio 1:10 until 1:20 is widely accepted as it visually does not interfere with the pathologist's impression on diagnosis finding. Automated tissue-based measurements and diagnostics are of a different nature when compared to human performance: of significance is a homogenous illumination in order to secure correct intensity measurement of detected objects and adequate texture analysis as well as a sufficient number of pixels covered by objects and sufficient image space to allowing the computation of reproducible texture algorithms.

Image quality analysis and implementation of standardized images for further image processing can be easily implemented into Grid technology [26] allowing worldwide access and promotion of computer assistance in diagnostic surgical pathology. Grid implementation in tissue-based diagnosis is still in its childhood; however expanding fast with correspondent progress in communication technology.

## References

[B1] Kayser K, Kayser G, Gu J, Ogilvie R (2005). Virtual Microscopy and Automated Diagnosis in Virtual Microscopy and Virtual Slides in Teaching, Diagnosis and Research.

[B2] Kayser K, Molnar B, Weinstein R (2006). Virtual Microscopy: Fundamentals, Applications, Perspectives of Electronic Tissue-based Diagnosis.

[B3] Kayser K, Radziszowski D, Bzdyl P, Sommer R, Kayser G (2006). Digitized pathology: theory and experiences in automated tissue-based virtual diagnosis. Rom J Morphol Embryol.

[B4] Kayser G, Radziszowski D, Bzdyl P, Sommer R, Kayser K (2006). Theory and implementation of an electronic, automated measurement system for images obtained from immunohistochemically stained slides. Anal Quant Cytol Histol.

[B5] Glatz-Krieger K, Glatz D, Mihatsch MJ (2006). Virtual microscopy: first applications. Pathologe.

[B6] Glatz-Krieger K, Spornitz U, Spatz A, Mihatsch MJ, Glatz D (2006). Factors to keep in mind when introducing virtual microscopy. Virchows Arch.

[B7] Kumar RK, Velan GM, Korell SO, Kandara M, Dee DR, Wakefield D (2004). Virtual microscopy for learning and assessment in pathology. J Pathol.

[B8] Schrader T, Niepage S, Leuthold T, Saeger K, Schluns K, Hufnagl P, Kayser K, Dietel M (2006). The diagnostic path, a useful visualisation tool in virtual microscopy. Diagn Pathol.

[B9] Tuominen VJ, Isola J (2007). The Application of JPEG2000 in Virtual Microscopy. J Digit Imaging.

[B10] Weinstein RS, Descour MR, Liang C, Barker G, Scott KM, Richter L, Krupinski EA, Bhattacharyya AK, Davis JR, Graham AR, Rennels M, Russum WC, Goodall JF, Zhou P, Olszak AG, Williams BH, Wyant JC, Bartels PH (2004). An array microscope for ultrarapid virtual slide processing and telepathology. Design, fabrication, and validation study. Hum Pathol.

[B11] Kayser K, Metze K, Radziszowski D, Amir-Hoshang S, Goldmann T, Kosjerina Z, Mireskandari M, Kayser G (2008). Texture and object related automated information analysis in histological still images of various organs. AQCH.

[B12] Kayser K, Radziszowski D, Bzdyl P, Sommer R, Kayser G (2006). Towards an automated virtual slide screening: theoretical considerations and practical experiences of automated tissue-based virtual diagnosis to be implemented in the Internet. Diagn Pathol.

[B13] Otsu N (1979). A threshold selection method from grey level histograms. IEEE Trans on Systems, Man, and Cybernetics.

[B14] Hardy W, Vance J, Jones K, Kokubo Y (1982). Digital image processing: a path to better pictures. Scan Electron Microsc.

[B15] Wang M, Zhou X, Li F, Huckins J, King RW, Wong ST (2008). Novel cell segmentation and online SVM for cell cycle phase identification in automated microscopy. Bioinformatics.

[B16] Braumann UD, Scherf N, Einenkel J, Horn LC, Wentzensen N, Loeffler M, Kuska JP (2007). Large histological serial sections for computational tissue volume reconstruction. Methods Inf Med.

[B17] Huisman A, Ploeger LS, Dullens HF, Jonges TN, Belien JA, Meijer GA, Poulin N, Grizzle WE, van Diest PJ (2007). Discrimination between benign and malignant prostate tissue using chromatin texture analysis in 3-D by confocal laser scanning microscopy. Prostate.

[B18] Kohler A, Bertrand D, Martens H, Hannesson K, Kirschner C, Ofstadm R (2007). Multivariate image analysis of a set of FTIR microspectroscopy images of aged bovine muscle tissue combining image and design information. Anal Bioanal Chem.

[B19] Ruiz T, Radermacher M (2006). Three-dimensional analysis of single particles by electron microscopy: sample preparation and data acquisition. Methods Mol Biol.

[B20] Hall PO van der, Kraan MC, Tak PP (2007). Quantitative image analysis of synovial tissue. Methods Mol Med.

[B21] Kayser K (2006). Introducing diagnostic pathology. Diagn Pathol.

[B22] Barker NJ, Zahurak M, Olson JL, Nadasdy T, Racusen LC, Hruban RH (1998). Digital imaging of black and white photomicrographs: impact of file size. Am J Surg Pathol.

[B23] Bernas T, Asem EK, Robinson JP, Rajwa B (2006). Compression of fluorescence microscopy images based on the signal-to-noise estimation. Microsc Res Tech.

[B24] Okumura A, Suzuki J, Furukawa I, Ono S, Ashihara T (1997). Signal analysis and compression performance evaluation of pathological microscopic images. IEEE Trans Med Imaging.

[B25] Pritt BS, Gibson PC, Cooper K (2003). Digital imaging guidelines for pathology: a proposal for general and academic use. Adv Anat Pathol.

[B26] Gortler J, Berghoff M, Kayser G, Kayser K (2006). Grid technology in tissue-based diagnosis: fundamentals and potential developments. Diagn Pathol.

